# An observational study of lower limb muscle imbalance assessment and gait analysis of badminton players

**DOI:** 10.3389/fbioe.2024.1439889

**Published:** 2024-10-29

**Authors:** Ping Huang, Wenxin Xu, Zeyi Bai, Lin Yu, Qichang Mei, Yaodong Gu

**Affiliations:** ^1^ Institute of Physical Education and Sport Science, Fujian Normal University, Fuzhou, China; ^2^ Faculty of Sports Science, Ningbo University, Ningbo, China; ^3^ Research Academy of Grand Health, Ningbo University, Ningbo, China

**Keywords:** badminton player, gait analysis, muscle imbalance, plantar pressure, symmetry index

## Abstract

**Purpose:**

The imbalance of muscle strength indicators has a negative impact on players. Lower limb muscle imbalance can cause gait abnormalities and increase the risk of muscle injury or decreased performance in significantly asymmetrical situations. This study aims to assess the lower limb muscle imbalance and gait feature between the dominant and non-dominant sides of badminton players and the associations between the two variables.

**Methods:**

The study included 15 badminton players with years of training experience. Muscle strength and gait parameters were obtained from isokinetic muscle strength testing and plantar pressure analysis systems. The symmetry index was calculated based on formulas such as plantar pressure distribution and percentage of plantar contact area.

**Results:**

In the isokinetic muscle strength test, significant differences were found in bilateral knee flexors’ average power and total work at 60°/s angular speed. The hamstring to quadriceps ratio (H/Q) range of knee joints of the dominant and non-dominant sides is 0.63–0.74 at low speed, while the H/Q range is 0.81–0.88 at fast speed. The H/Q of bilateral knees increases with increasing angular velocity. As the angular velocity increases, the peak torque to body weight ratio (PT/BW) of the participants’ bilateral knee flexors and extensors shows a decreasing trend. The asymmetry score of H/Q at 180°/s angular speed is positively related with step time and stance time. There are varying degrees of differences in gait staging parameters, plantar pressure in each area, plantar contact area, and symmetry index between the dominant and non-dominant sides of badminton players when walking.

**Conclusion:**

Badminton players have weaker flexors of the knee joint, imbalanced muscle strength in flexors and extensors, decreased lower limb stability, and a risk of knee joint injury on the non-dominant side. The bending and stretching strength of the knee joint on the dominant side of the players is greater than that on the non-dominant side. The pressure in the first metatarsal region of the dominant side is higher, while that in the midfoot and heel regions is higher on the non-dominant side. badminton players have better forward foot force and heel cushioning ability. Long term badminton sports result in specialized changes in plantar pressure distribution and reduced symmetry.

## 1 Introduction

Symmetry emerged through natural selection in evolution and has the characteristic of simple coding, and the human body is usually considered a biological structure with symmetry (Johnston et al., 2022). However, the reduced multitasking ability, high energy consumption, and bilateral action control issues result in asymmetric development of the nervous system (Ocklenburg and Mundorf, 2022). In human daily activities, long-term specialized training and abnormal movement patterns caused by injuries and illnesses lead to structural and functional abnormalities. The difference in the structure and function of bilateral limbs is called limb asymmetry (Bishop et al., 2018; Keeley et al., 2011). Players in sports such as football (Croisier et al., 2008), softball (Newton et al., 2006), and speed skating (Shu et al., 2023) all suffer from limb asymmetry. Badminton, as a handheld racket sport, has been proven to cause upper limb muscle imbalance (Gasibat et al., 2023). The lower limb lunge movement, repeated starts, sudden stops, and changing direction in badminton cause rapid changes in joint angles of the lower limb, as well as the larger ground reaction force during takeoff and landing, resulting in lower limb asymmetry, which is also the leading cause of lower limb injuries (Wei, 2009; Shen et al., 2024). The body rebuilds muscle balance through compensation within an acceptable load range to complete basic technical movements. The impact on the body is not apparent in the short term. However, abnormal movement patterns can occur when the accumulation exceeds the body’s limit, leading to increased fatigue or injury (Grindem et al., 2011; Yu et al., 2023a). Therefore, it is important to evaluate the imbalance of lower limb muscle strength indicators in badminton players.

Muscle imbalance can be assessed by the differences between agonist and antagonists and the bilateral differences (Gioftsidou et al., 2008; Henderson et al., 2010). The characteristics of repeated lunges and rapid return in badminton result in differences in the strength of the knee flexors and extensors, which are believed to be related to hamstring injury (Chin et al., 1994; Askling et al., 2003; Orchard et al., 1997). The relatively weak hamstring muscle may be one of the reasons for the increased incidence of anterior cruciate ligament injury during this sport (Russell et al., 1995). Reliable and repeatable measuring tools can identify abnormal patterns in the body (Safayi et al., 2015; Krizsan-Agbas et al., 2014). When comparing the strength ratios of the hamstring and quadriceps muscles, isokinetic strength testing is commonly used for identifying imbalances (Bennell et al., 1998; Askling et al., 2003). Muscle imbalance and lateral movement in the lunge cause abnormal gait in badminton players. Gait analysis is a method to monitor walking patterns through experimental instruments, which can objectively evaluate the functional behavior of the feet and reveal essential aspects and influencing factors of gait abnormalities (Klöpfer-Krämer et al., 2020; Yu et al., 2022). Plantar pressure distribution in normal individuals exhibits bilateral symmetry (Yuan et al., 2004) and is not affected by age, gender, or weight (Luger et al., 1999). Due to repeated practice of specialized movements, players develop a characteristic distribution of specialized plantar pressure. Analyzing the distribution and changes of plantar pressure in specialized players and then analyze abnormal gait, providing a valuable reference for evaluating lower limb function in specialized players. Understanding the characteristics of plantar pressure distribution can effectively optimize technical movements, reduce foot injuries, and improve the design of specialized shoes (Morlock and Nigg, 1991; Zhang and Ni, 2012).

It is important to understand the muscle imbalance of badminton players, because it can better guide training arrangements and provide a reference for developing professional badminton footwear (Gasibat et al., 2023). This study aims to evaluate the differences in lower limb muscle strength between the dominant and non-dominant sides, as well as the differences between the flexor and extensor muscles in players who have engaged in long-term badminton training. Furthermore, the study seeks to determine the presence of muscle balance. The differences in dynamic plantar pressure parameters, gait cycle characteristics, and symmetry index of participants during walking were evaluated using a plantar pressure system.

## 2 Methods

### 2.1 Participant characteristics and body composition assessments

A total of 15 advanced skill male badminton players from the school team (age: 20.8 ± 2.3 years, height: 174.47 ± 5.3 cm, weight: 67.79 ± 6.87 kg, years of playing badminton: 5.1 ± 1.8 years) participated in this study. All participants held the racket with their right hand, thus the right side was defined as the dominant side. Prior to testing, a body composition assessment was conducted using the Tanita MC-180. The muscle masses of left upper limb, right upper limb, left lower limb and right lower limb were 2.57 ± 0.33 kg, 2.89 ± 0.25 kg, 10.81 ± 0.95 kg and 11.03 ± 1.04 kg, respectively, with greater muscle mass observed on the right side compared to the left. All participants were confirmed to have normal anatomical structure and function, good physical condition, and motor ability with no acute or chronic injuries to their lower limbs and feet, as verified through a medical history inquiry and physical examination. All participants voluntarily signed an informed consent form before the experiment. This study was approved by the Institutional Review Board of Ningbo university (TY2024007), in strict accordance with the principles outlined in the 1975 Helsinki Declaration.

### 2.2 Lower limb muscle strength measurement

The CON-TREX device (CON-TREX MJ Multi-joint Module, CMVAG, Switzerland) was used to assess the strength of antagonistic muscle groups in the knee joint. Prior to testing, the participants were familiarized with the experimental procedure and underwent a thorough warm-up. The testing instruments were calibrated routinely. The side holding the racket was defined as the dominant side and the opposite side was the non-dominant side. Testing strictly followed the methods specified in the experimental instrument manual, using the centripetal mode and following the isokinetic testing standard developed by Davies and Ellenbecker, (2012). Joint testing velocities of 60°/s and 180°/s were selected. 1-min interval between groups and 3∼5-min interval between both sides were taken during testing. The analyzed parameters included peak torque (PT) (Nm, maximum torque of muscles or joints), average power (AP) (J/s, muscle work per unit time), total work (TW) (J, product of force and angular displacement), peak torque to body weight ratio (PT/BW) (Nm/kg, muscle torque per unit weight), and the hamstring to quadriceps ratio (H/Q) (%, strength ratio between agonist and antagonist muscle groups). The left and right strength scores were normalized and expressed as a percentage: Asymmetry = |mean R - mean L|/mean [R, L] * 100.

### 2.3 Gait assessment

**FIGURE 1:**
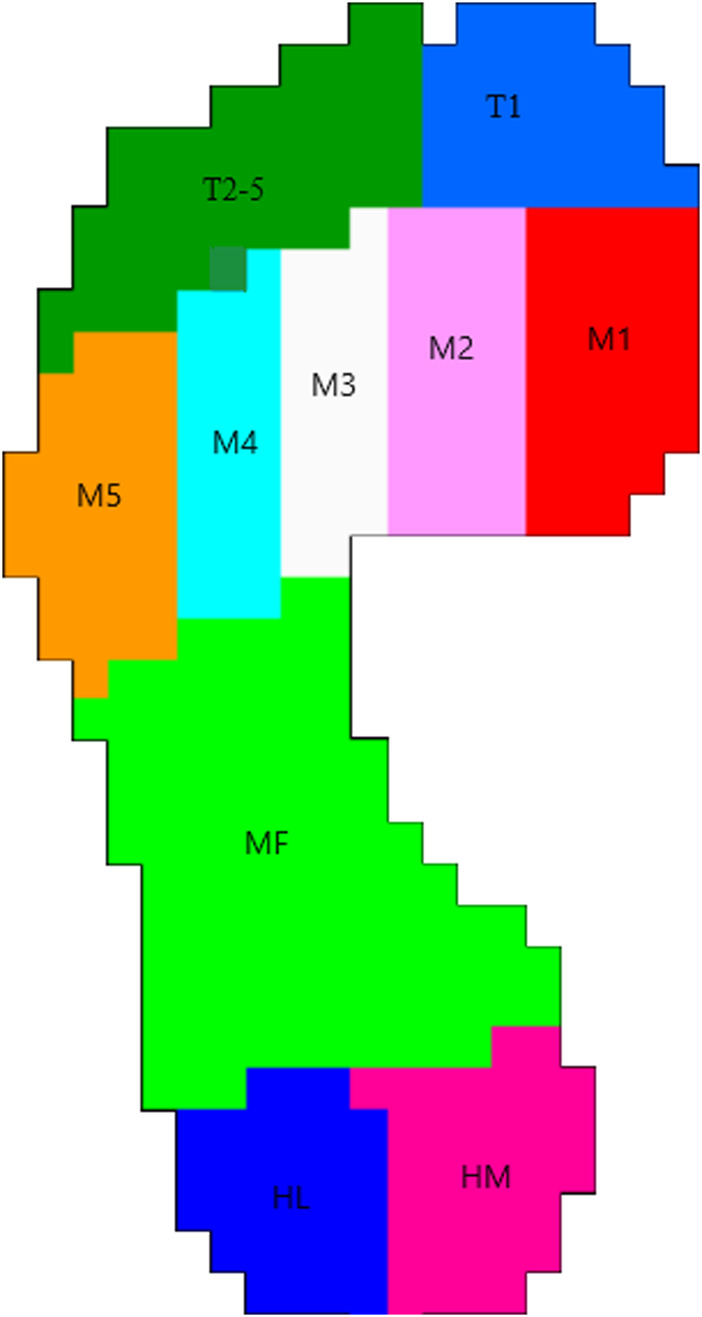
Ten anatomical regions of plantar pressure.

Plantar pressure during walking was assessed using the Foot-scan plate system (RS Scan International, Belgium), consisting of a 2 m × 0.5 m flat plate, with a 3-m-long, equally wide floor mat placed before and after the plate. Trained professionals conducted the test. Prior to testing, the surveyors demonstrated the measurement method to the participants and explained the necessary precautions. First, participants were instructed to remove their shoes and socks for a barefoot test. They were then given time to walk adaptively. Subsequently, the participants were asked to walk naturally, without any restrictions on stride length or pace, ensuring they avoided walking around the edges of the plate. Each measurement was repeated five times to ensure the collection of reliable data. The test parameters for gait feature analysis included gait velocity (m/s), step time (seconds), stance time (seconds) and double support phase (percentage of gait cycle time). The test parameters for plantar pressure analysis were measured in 10 anatomical regions (Figure 1): toe 1 (T1), toes 2–5 (T2-5), first metatarsal head (M1), second metatarsal head (M2), third metatarsal head (M3), fourth metatarsal head (M4), fifth metatarsal head (M5), midfoot (MF), heel medial (HM), and heel lateral (HL). The symmetry index (SI) of gait variables, including peak pressure and contact area, was calculated according to Tyrell et al. (Tyrell et al., 2011) and Hsu et al. (Hsu et al., 2003). An SI value of 0 indicates perfect symmetry.

### 2.4 Statistical Analyses

Statistical analysis was performed by using SPSS 24.0 (IBM Corporation, Armonk, NY). Continuous data was expressed by mean ± standard deviation (M ± SD). The Mann-Whitney U test was applied to compare gait phase parameters with a non-normal distribution between the bilateral lower limbs, while an independent-samples t-test was used to analyze differences in plantar pressure and muscle strength between the bilateral lower limbs. Statistical significance was set at *p* < 0.05 (*). Pearson correlation coefficient was used to evaluate the relationship between lower limb muscle strength data and gait features. The coefficient was classified using the following definitions: 0–0.19, very weak; 0.20–0.39, weak; 0.40–0.59, moderate; 0.60–0.79, strong; 0.80–1.00, very strong.

## 3 Results

This study mainly focused on the lower limb muscle imbalance and gait features of badminton players. Parameters such as bilateral lower limb muscle strength distance and average power were obtained through isokinetic muscle strength testing, and the knee joint H/Q was calculated to evaluate the balance of unilateral flexion extension muscle group. Weight standardization of peak torque was used for comparison between different individuals. Comparison of lower limb muscle strength and gait data between dominant and non-dominant sides was used to evaluate lower limb asymmetry.

### 3.1 Isokinetic muscle strength test results

Table 1 shows that at 60°/s, significant differences were found in the flexors’ average power and total work and in the H/Q of the flexors and extensors between bilateral knee joints (*p* < 0.01). In addition, at different angular velocities, there was no significant difference in the peak torque of flexors and extensors between bilateral knee joints. The dominant side was higher than the non-dominant side. The strength ratio of the flexors and extensors was 0.63–0.74 at 60°/s but significantly increased to 0.81–0.88 at fast speed (*p* < 0.05).

**TABLE 1:** Characteristics of muscle strength of bilateral knee joint (X ± s, n = 15).

			Non-dominant side	Dominant side	T	P	Strength asymmetry score
Peak torque [Nm]	60°/s	Extensors	152.77 ± 22.42	154.47 ± 36.46	−0.15	0.88	4.14 ± 2.10
		Flexors	107.23 ± 11.03	113.58 ± 20.78	−0.98	0.34	13.09 ± 6.98
	180°/s	Extensors	122.45 ± 25.54	142.18 ± 26.27	−1.91	0.07	18.27 ± 8.41
		Flexors	105.44 ± 25.65	121.18 ± 28.30	−1.54	0.14	21.36 ± 10.65
Average power [w]	60°/s	Extensors	76.81 ± 13.86	74.57 ± 22.77	0.32	0.76	17.44 ± 8.63
		Flexors	53.45 ± 11.24	65.65 ± 18.76	−2.12	0.05*	14.54 ± 3.69
	180°/s	Extensors	140.68 ± 29.16	146.29 ± 34.13	−0.46	0.65	12.28 ± 7.90
		Flexors	104.18 ± 21.43	117.56 ± 24.53	−1.48	0.15	15.98 ± 7.54
Total work [J]	60°/s	Extensors	525.98 ± 114.23	559.47 ± 133.74	−0.68	0.51	12.58 ± 5.28
		Flexors	387.17 ± 84.93	457.87 ± 75.02	−2.16	0.04*	22.79 ± 2.14
	180°/s	Extensors	426.30 ± 111.73	435.34 ± 143.89	−0.19	0.85	16.23 ± 8.94
		Flexors	332.56 ± 80.21	375.51 ± 88.70	−1.34	0.19	15.94 ± 9.26
Hamstring to quadriceps ratio (H/Q) [%]	60°/s		0.63 ± 0.11	0.74 ± 0.13	−2.86	0.01*	17.70 ± 10.94
	180°/s		0.81 ± 0.12*	0.88 ± 0.10*	−1.94	0.06	10.52 ± 3.31

Note: * represents relative to 60°/s angular speed; the H/Q of the flexors and extensors significantly increased at 180°/s (*p* < 0.05).

Table 2 shows that PT/BW of the dominant and non-dominant sides decreases with increasing angular velocity, and extension is greater than flexion at different angular velocities. At 60°/s angular speed, the non-dominant knee joint’s flexion and extension PT/BW were significantly greater than at 180°/s. In contrast, flexion PT/BW of the dominant knee joint was significantly greater than at 180°/s.

**TABLE 2:** Results of relative peak torque at different angular velocities (X ± s).

	Non-dominant side		Dominant side		Strength asymmetry score	
	Extensors	Flexors	Extensors	Flexors	Extensors	Flexors
60°/s	1.86 ± 0.38	1.09 ± 0.39	1.96 ± 0.54	1.31 ± 0.48	12.63 ± 6.81	16.03 ± 10.20
180°/s	1.50 ± 0.46*	0.75 ± 0.46*	1.60 ± 0.54	0.83 ± 0.43*	22.10 ± 11.60	12.08 ± 4.95

Note: PT/BW (N· m/kg), * represents relative to 60°/s angular speed; the difference was significant at 180°/s (*p* < 0.05).

### 3.2 Dynamic characteristics

#### 3.2.1 Distribution of plantar pressure on the dominant and non-dominant side

**FIGURE 2:**
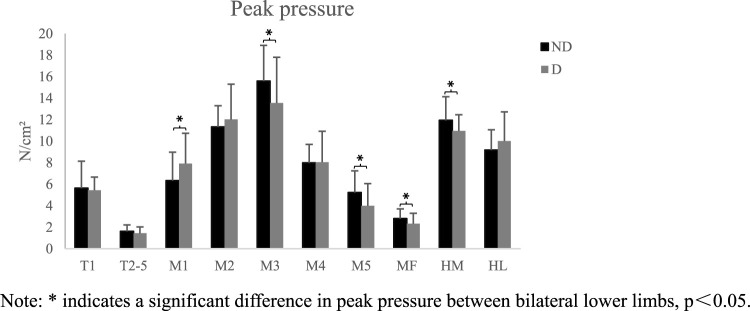
Schematic diagram of peak pressure in each area (X ± s). Note: * indicates a significant difference in peak pressure between bilateral lower limbs, p<0.05.

**FIGURE 3:**
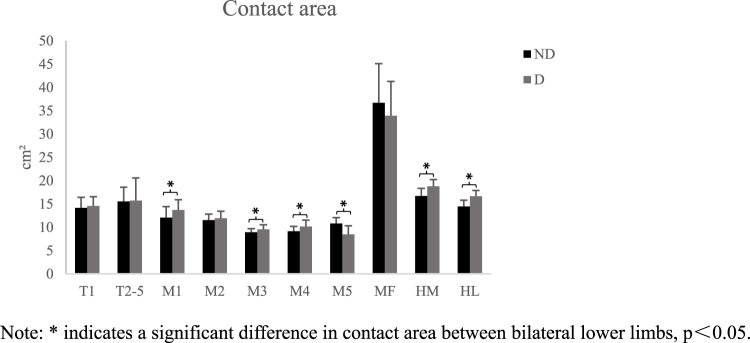
Schematic diagram of contact area in each area (X ± s). Note: * indicates a significant difference in contact area between bilateral lower limbs, p<0.05.

During walking, differences were observed in peak pressure across the ten regions of the plantar area of the participants (Figure 2). Peak pressure was highest in the second and third metatarsal regions, while the areas with lower peak pressure were mainly located in the T2-5 and MF regions. The peak pressure of the medial forefoot on the dominant side was higher than that on the non-dominant side, with a significant difference in the M1 area (*p* < 0.05). The peak pressure of the midfoot and heel medial on the non-dominant side was significantly higher than on the dominant side (*p* < 0.05). Additionally, significant differences were found in the contact area (Figure 3). The contact area of the dominant side was significantly larger than that of the non-dominant side in the first-fifth metatarsal and heel regions (*p* < 0.05). The SI of pick pressure was 0.18 ± 0.11, and that of contact area was 0.14 ± 0.11.

#### 3.2.2 Gait phase parameters

**FIGURE 4:**
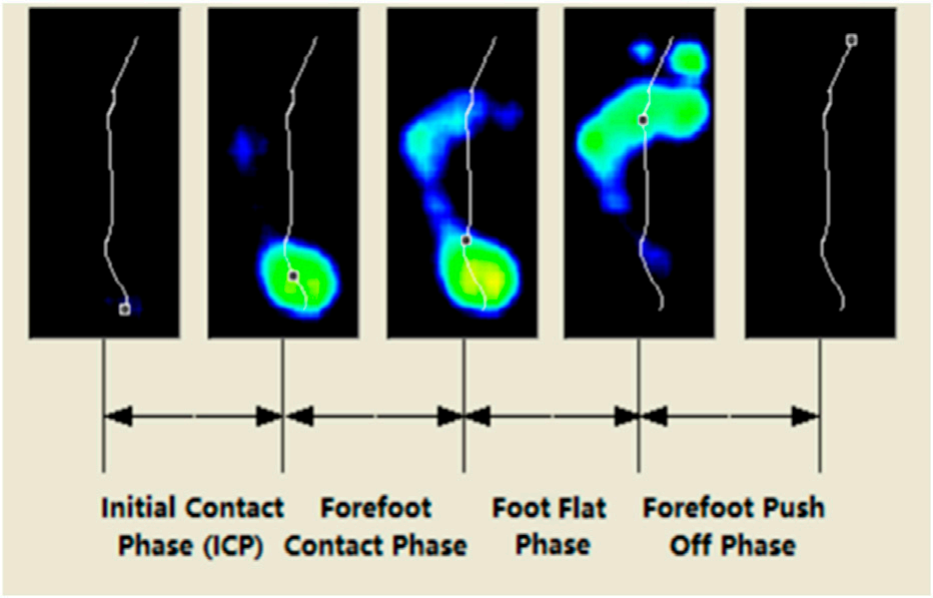
Phases of the gait cycle.

Walking is a cyclical movement. The period from when one heel lifts off the ground until it contacts the ground again is referred to as a gait cycle. A gait cycle includes a stance phase and a swing phase. This experiment divided the gait cycle into heel strike, pre-midstance, midstance, and propulsion (Figure 4). The Gait phase parameters are expressed as the percentage of each time phase to the total time. There was no significant difference between the dominant and non-dominant feet of the participants at different time phases, but the dominant side had a longer landing time on the forefoot than the non-dominant side, and the non-dominant side required more time in the final propulsion phase (See Table 3).

**TABLE 3:** Comparison of gait phase parameters among participants.

Phase	Non-dominant side	Dominant side	Z	P
Heel strike (%)	8 (6.5, 9)	8 (7, 10)	−1.09	0.28
Pre-Midstance (%)	33.5 (28.25, 40)	36 (25, 41)	−0.53	0.59
Midstance (%)	8 (6, 10)	7 (4, 11)	−1.23	0.22
Propuls*ion (%)	48.5 (42.25, 53.0)	46.5 (41.0, 55.25)	−0.36	0.72

**FIGURE 5:**
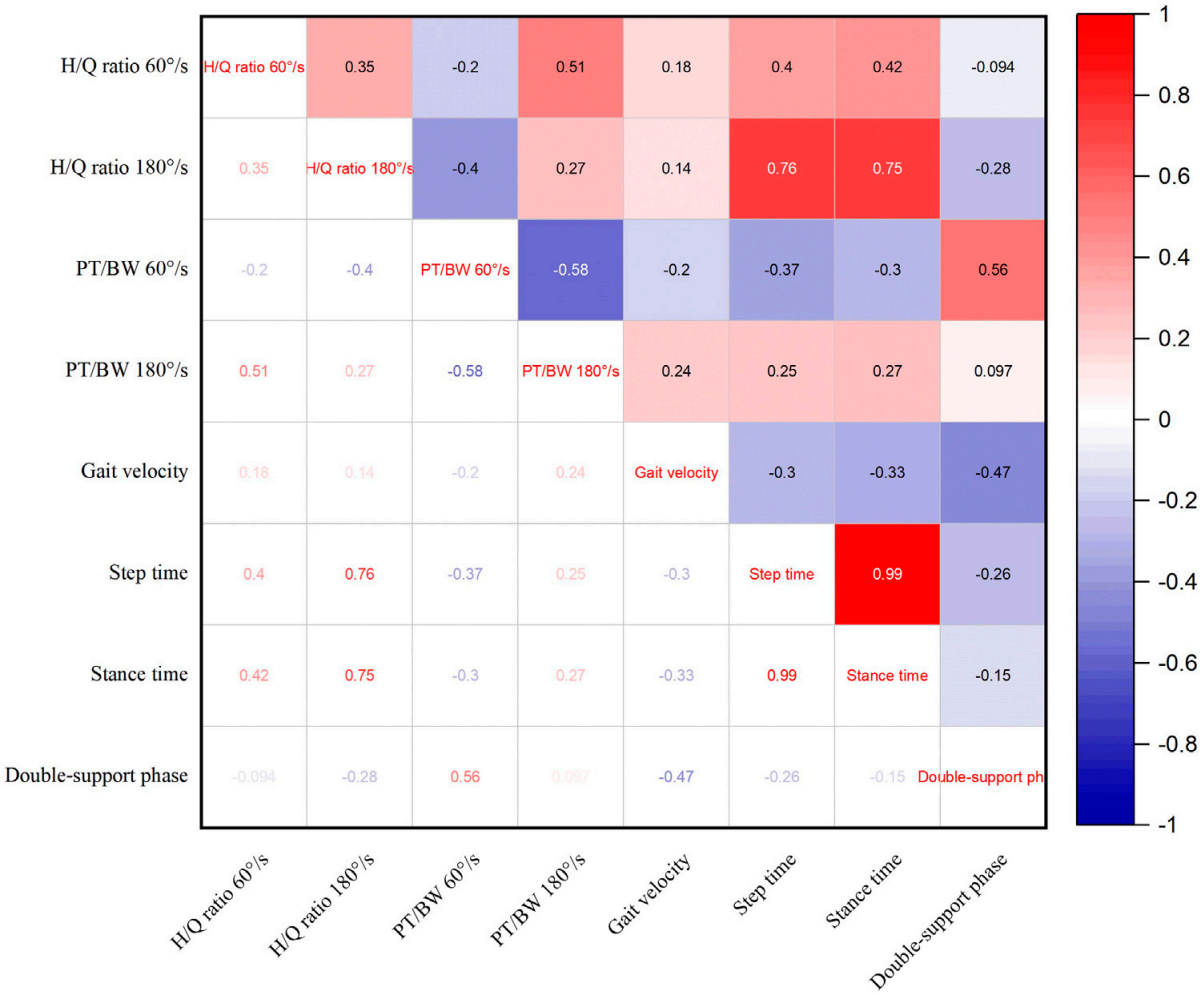
Correlation analysis between lower limb muscle strength and gait features.

Figure 5 shows the correlation analysis between lower limb muscle strength and gait features. The lower limb muscle strength parameters were asymmetry scores of H/Q and PT/BW at speeds of 60°/s and 180°/s. The gait parameters included gait velocity, step time, stance time and double support phase. The results show that the asymmetry score of lower limb H/Q ratio at 180°/s is positively correlated with step time and stance time.

## 4 Discussion

Bilateral lower limb asymmetry and muscle imbalance are influencing factors of sports injuries (Dauty et al., 2016; Hiemstra et al., 2004). Traditional views suggest that reducing limb asymmetry can reduce the risk of injury (Askling et al., 2003; Croisier et al., 2002; Orchard et al., 1997). Methods of identification and calculation for lower limb functional asymmetry should be selected based on sport-specific characteristics in order to have a scientific understanding of the impact of lower limb functional asymmetry on players. This study analyzed the lower limb muscle function of badminton players through dynamic analysis methods, providing a basis for targeted strength training and injury prevention in badminton.

Asymmetry on the dominant and non-dominant sides is common among badminton players due to the specific characteristics of the sport. This study found that when comparing the flexors of bilateral knee joints in players, significant differences were found in the average power and total work at 60°/s. This is related to the footwork characteristics of badminton. In the middle and front court movement, the dominant side completes the lunge movement. During the lunge, the muscle strength of the flexors and extensors of the dominant side is enhanced, while the non-dominant side usually maintains body balance, resulting in weaker flexors on the non-dominant side. The knee joint H/Q represents the balance of muscle strength between agonist and antagonist muscle groups, indirectly reflecting the stability of the knee joint (Li, 2022). The results of this study showed that at low speed, the H/Q range of the dominant and non-dominant knee joints was 0.63–0.74, while at fast speed, the H/Q range was 0.81–0.88. There is a significant difference in the H/Q of the flexors and extensors between the bilateral sides at 60°/s. Previous studies found that the H/Q range of the knee joint is generally from 50% to 80% (Grace et al., 1984; Raunest et al., 1996; Bennell et al., 1998). Experimental studies have shown that the bilateral knee joint H/Q of young national male badminton athletes is within normal values, which is of great significance for maintaining stability (Liang et al., 2021). Therefore, college badminton players should strengthen the strength of the knee joint flexors. Otherwise, knee joint injuries may occur during explosive power output. There were significant differences between H/Q at 60°/s and that at 180°/s both on the dominant side and non-dominant side, indicating that as badminton players increase their movement speed or the intensity of the game increases, the contribution of the posterior thigh muscle during lunge increases, thereby achieving the requirements of rapid brace and return. PT/BW can standardize body weight and compare differences between different individuals. The results of this study showed that as the angular velocity increased, the flexion and extension PT/BW of bilateral knee joints in the participants showed a decreasing trend, which is consistent with the variation law of the muscle force-velocity curve. When the dominant side is in the middle or front court for a lunge, the non-dominant side usually quickly flexes and follows. Previous studies have suggested that athletes with higher levels of skill typically have stronger flexion abilities on the non-dominant side, but further research is needed (Liang et al., 2021). The results of this study support the hypothesis that college badminton players with years of training experience have weaker flexion ability on the non-dominant knee joint.

The anatomical features of ordinary people are approximately symmetrical. Therefore, gait characteristics and muscle activation in motion control are symmetrical. The highest plantar pressure in healthy adults during walking is in the third metatarsal bone, followed by the heel (Periyasamy et al., 2011; Soames, 1985; Hsu et al., 2003), which is consistent with the distribution trend of plantar pressure in this study. When comparing the peak pressure on the dominant and non-dominant sides, the pressure in the first metatarsal region on the dominant side is significantly higher than that on the non-dominant side, whereas the pressure in the middle and outer regions of the forefoot on the dominant side is significantly lower than that on the non-dominant side. Badminton players experience greater pressure in the first metatarsal area of their dominant side during the starting, braking, and returning phases, especially in the main force-producing area in the medial forefoot during the returning phase (Yu et al., 2023b). Therefore, when designing badminton footwear, stress concentration in the first metatarsal area should be considered more. The pressure in the midfoot and heel on the non-dominant side is significantly higher than that on the dominant side because in badminton footwork, most of the force is generated by the forefoot propulsion, and the dominant side dominates the forward driving force. The pressure in the metatarsal and heel regions on the dominant side is significantly different from that on the non-dominant side, indicating that athletes who have undergone years of badminton training can reduce the risk of injury and provide more stable support by increasing the contact area of the foot to buffer local pressure.

Limb asymmetry during walking is considered an important variable for evaluating gait quality (Awad et al., 2015; Tyrell et al., 2011; Hsu et al., 2003). The research results indicate that there is an asymmetry in plantar pressure and contact area. The asymmetrical gait of athletes who engage in badminton for the long term is mainly caused by muscle imbalance. In training practice, limb asymmetry should be combined with basic strength, explosive power, and other assessments to optimize the evaluation system of advanced skill athletes and provide new tools for a deeper understanding of athlete physical deficiencies. The results of the gait phase parameters suggest that there is no significant difference between the bilateral limbs of the participants at each time phase, but the landing time of the dominant side is longer than that of the non-dominant side, while the final propulsion time of the dominant side is shorter than that of the non-dominant side. Badminton players who use their dominant side more frequently may exhibit longer forefoot landing times, and this preference may lead to adaptive changes in muscles and nervous systems. The shorter propulsion time of the dominant side is also related to forefoot propulsion in the starting phase of badminton footwork. Correlation analysis between asymmetry scores of lower limb muscle strength and gait features found that the asymmetry score of H/Q ratio at 180°/s was positively correlated with step time and stance time. It reflects that badminton players adapt to asymmetrical strength by adjusting their gait. This adjustment may be due to the enhancement of the weak side stability through compensation mechanism of body by extending step time. When there is a significant difference in H/Q ratio, players need longer stance time to maintain balance, leading to a decrease in dynamic control ability caused by power asymmetry. However, the limitation of this study is the small sample size. In future research, the gait characteristics of ordinary people can be added for comparative analysis of the non-equilibrium characteristics generated by long-term badminton sports.

## 5 Conclusion

College badminton players have weaker knee flexors on the non-dominant side, which is significantly different from advanced skill athletes. Therefore, it is necessary to enhance the specialized strength of the flexors during training and competition. Given the H/Q exceeds the normal range at 180°/s, the explosive training of the flexors and extensors should be strengthened to reduce the risk of a knee injury. During walking, the first metatarsal bone experiences greater pressure, which should be considered in shoe design. In the gait phase parameters, athletes who have undergone long-term badminton training have better adaptability to changes in buffering and propulsion force on the dominant side.

Flexor training should be strengthened to reduce muscle imbalance in the flexors and extensors, therefore enhancing knee joint stability. In traditional training modes, the strength imbalance of bilateral limbs still exists during bilateral strength exercises such as squats. The sport of holding the racket on one side makes these imbalances permanent, and specific resistance training may be required for the weaker side.

## Data Availability

The raw data supporting the conclusions of this article will be made available by the authors, without undue reservation.
