# Supplementary material for: An observational study of lower limb muscle imbalance assessment and gait analysis of badminton players
Source: Front Bioeng Biotechnol. 2024 Oct 29;12:1439889. doi: 10.3389/fbioe.2024.1439889 (PMC11554494; doi:10.3389/fbioe.2024.1439889)
Supplement: Supplementary file 1 [file DataSheet1.docx]

Supplementary Material

# Supplementary Figures and Tables

## Supplementary Figures


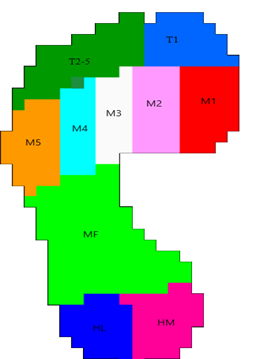


**Supplementary Figure 1.** Ten anatomical regions of plantar pressure.

**Supplementary Figure2.** Schematic diagram of peak pressure in each area (X±s)

**Supplementary Figure 3.** Schematic diagram of contact area in each area (X±s)


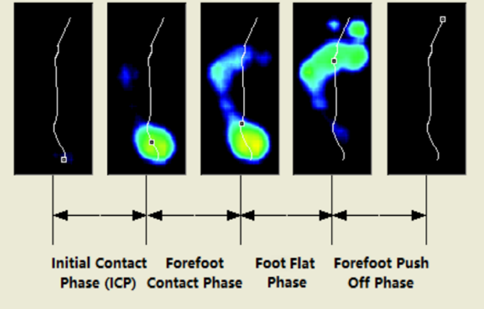


**Supplementary Figure 4.** Phases of the gait cycle


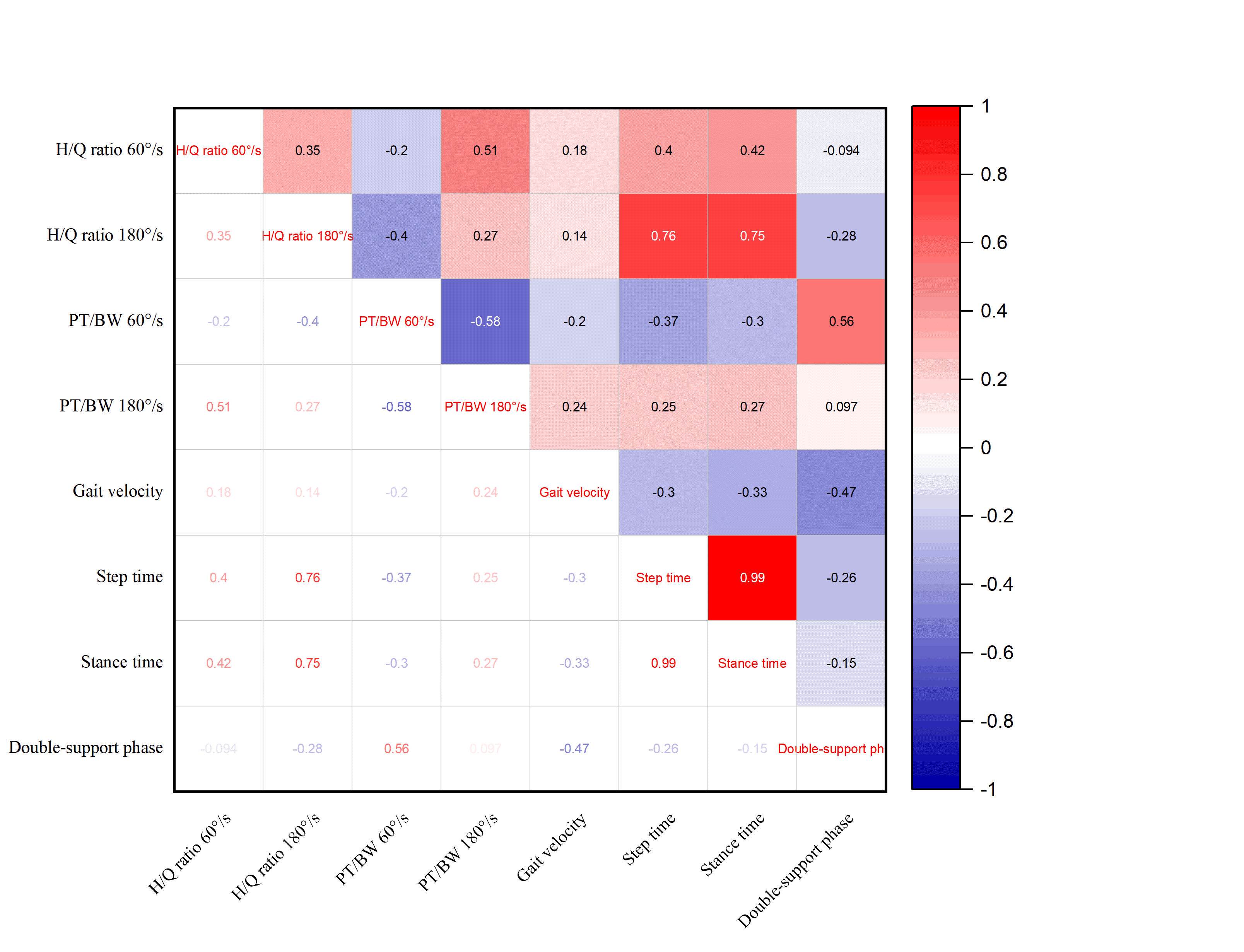


**Supplementary Figure 5.** Correlation analysis between lower limb muscle strength and gait features

## Supplementary Tables

**Table 1. Characteristics of muscle strength of bilateral knee joint (X±s，n=15)**

|  |  | | Non-dominant side | Dominant side | T | P | Strength asymmetry score |
| --- | --- | --- | --- | --- | --- | --- | --- |
| Peak torque [Nm] | 60˚/s | Extensors | 152.77±22.42 | 154.47±36.46 | -0.15 | 0.88 | 4.14±2.10 |
|  |  | Flexors | 107.23±11.03 | 113.58±20.78 | -0.98 | 0.34 | 13.09±6.98 |
|  | 180˚/s | Extensors | 122.45±25.54 | 142.18±26.27 | -1.91 | 0.07 | 18.27±8.41 |
|  |  | Flexors | 105.44±25.65 | 121.18±28.30 | -1.54 | 0.14 | 21.36±10.65 |
| Average power [w] | 60˚/s | Extensors | 76.81±13.86 | 74.57±22.77 | 0.32 | 0.76 | 17.44±8.63 |
|  |  | Flexors | 53.45±11.24 | 65.65±18.76 | -2.12 | 0.05* | 14.54±3.69 |
|  | 180˚/s | Extensors | 140.68±29.16 | 146.29±34.13 | -0.46 | 0.65 | 12.28±7.90 |
|  |  | Flexors | 104.18±21.43 | 117.56±24.53 | -1.48 | 0.15 | 15.98±7.54 |
| Total work [J] | 60˚/s | Extensors | 525.98±114.23 | 559.47±133.74 | -0.68 | 0.51 | 12.58±5.28 |
|  |  | Flexors | 387.17±84.93 | 457.87±75.02 | -2.16 | 0.04* | 22.79±2.14 |
|  | 180˚/s | Extensors | 426.30±111.73 | 435.34±143.89 | -0.19 | 0.85 | 16.23±8.94 |
|  |  | Flexors | 332.56±80.21 | 375.51±88.70 | -1.34 | 0.19 | 15.94±9.26 |
| Hamstring to quadriceps ratio(H/Q) [%] | 60˚/s |  | 0.63±0.11 | 0.74±0.13 | -2.86 | 0.01* | 17.70±10.94 |
|  | 180˚/s |  | 0.81±0.12* | 0.88±0.10* | -1.94 | 0.06 | 10.52±3.31 |

Note: * represents relative to 60˚/ s angular speed; the H/Q of the flexors and extensors significantly increased at 180˚/ s (p<0.05).

**Table 2. Results of relative peak torque at different angular velocities (X±s)**

|  | Non-dominant side | | Dominant side | | Strength asymmetry score | |
| --- | --- | --- | --- | --- | --- | --- |
|  | Extensors | Flexors | Extensors | Flexors | Extensors | Flexors |
| 60˚/s | 1.86±0.38 | 1.09±0.39 | 1.96±0.54 | 1.31±0.48 | 12.63±6.81 | 16.03±10.20 |
| 180˚/s | 1.50±0.46* | 0.75±0.46* | 1.60±0.54 | 0.83±0.43* | 22.10±11.60 | 12.08±4.95 |

Note: PT/BW (N· m/kg), * represents relative to 60˚/s angular speed; the difference was significant at 180˚/s (p<0.05).

**Table 3. Comparison of gait phase parameters among participants**

| Phase | Non-dominant side | Dominant side | Z | P |
| --- | --- | --- | --- | --- |
| Heel strike (%) | 8 (6.5, 9) | 8 (7, 10) | -1.09 | 0.28 |
| Pre-Midstance (%) | 33.5 (28.25, 40) | 36 (25, 41) | -0.53 | 0.59 |
| Midstance (%) | 8 (6, 10) | 7 (4, 11) | -1.23 | 0.22 |
| Propuls*ion (%) | 48.5 (42.25, 53.0) | 46.5 (41.0, 55.25) | -0.36 | 0.72 |
